# Psychological Functioning, Defense Strategies and the Contribution of Perceived Family Collaboration in Adolescents Who Experienced Multiple Motor Vehicle Crashes: A Descriptive Study

**DOI:** 10.3390/ijerph19159448

**Published:** 2022-08-02

**Authors:** Paola Di Vito, Luca Cerniglia, Silvia Cimino

**Affiliations:** 1 Department of Dynamic and Clinical Psychology, University of Rome, Sapienza, 00186 Rome, Italy; divito.1699290@studenti.uniroma1.it (P.D.V.); silvia.cimino@uniroma1.it (S.C.); 2 Faculty of Psychology, International Telematic University Uninettuno, 00186 Rome, Italy

**Keywords:** adolescence, motor vehicle accidents, family functioning, emotional–behavioral functioning, alexithymia, defense strategies

## Abstract

Adolescents frequently experience motor vehicle collisions (MVCs). Research has mostly underlined the psychological outcomes, rather than adolescents’ general emotional–behavioral functioning and the role played by family. This study aims to explore the emotional–behavioral functioning, measured with the Youth Self Report (YSR), difficulties to identify and describe emotions, measured with the Toronto Alexithymia Scale (TAS-20), the use of defense strategies, measured with the Response Evaluation Measure for Youth (REM-71), and perceived family collaboration, measured with the Perceived Collective Family scale, in adolescents that have experienced more than three MVCs in a year. N = 150 adolescents who visited an emergency department for MVCs were assessed through self-report questionnaires. Adolescents showed difficulties to identify and describe their emotions and a massive use of defense strategies. Moreover, lower perceived family collaboration predicted adolescents’ alexithymic traits and the massive use of maladaptive defense strategies. These results may be useful in assessing and creating prevention programs for risky driving behaviors in adolescence.

## 1. Introduction

As shown by several studies, the possibility of experiencing a traffic collision is three times greater for adolescents compared to older drivers [1]. In 2019, nearly 2400 teens aged 13 to 19 were killed in the United States and roughly 250,000 were transported to emergency rooms for injuries sustained in road and traffic accidents [2]. Specifically, in Italy, motor vehicle collisions (MVCs) are the first cause of death in people under 30 years of age. Of injuries, 67% involve youth riding motorcycles [3]. The risk of a collision is higher in adolescents aged 16 to 19 compared to any other age group. Teen drivers and passengers in this age group are almost three times more likely to be involved in a mortal collision than older drivers (20 yrs old or more). There is a vast literature that addresses the role of distraction in traffic collisions. Driving behavior and practices are some of the most crucial factors in traffic collisions [4] and the use of mobile phones has been identified as one of the most distractive factors [5]. Driver distraction impairs risk perception and vehicle performance [6]. The National Household Travel Survey (2017) has identified other common risk factors for road collisions. Teens driving with other teens or young adult passengers are particularly at risk as newly licensed teen drivers. In fact, the risk of being involved in these types of collisions is especially high during the first months of driving [7]. Moreover, gender seems to play an important role in shaping the possibility of an MVC. In 2019, in the US, the number of traffic fatalities among male drivers aged 16 to 19 was more than twice that of female drivers of the same age. Male adolescents seem to show more driver aggression, sensation seeking, and general risk-taking compared to females of the same age [8]. Data from the National Household Travel Survey (2016–2017) show that the crash rate is 1.5 times higher for 16-year-old drivers than for 18- and 19-year-old drivers per mile driven. Inexperience is indicated as a potential risk factor. This is especially relevant in adolescence as driving inexperience positively affects risky driving behaviors mediated by sensation seeking and risk perception [9]. In fact, teens—more than older people—are prone to underrate or fail to identify perilous situations [8]. Risk-taking dramatically increases between childhood and adolescence due to the brain’s socioemotional remodeling of the dopaminergic system, leading to an increased desire for reward-seeking situations, more so when peers are present [10]. Another risk factor indicated by the Centers for Disease Control and Prevention [2] is driving at night and on weekends. This appears to be riskier than driving during the day in general, but it is especially dangerous for adolescent drivers. Adolescents and young adults are reported to have the lowest seat belt use. For example, results from the 2016–2019 National Occupant Protection Use Survey (NOPUS) Controlled Intersection Study show that the front seat belt use rate for teens and young adults (ages 16–24) was about 87% each year, while the front seat belt use rate for adults (ages 25 or older) was more than 90%. Notwithstanding the importance of these factors, research also suggests that traffic collisions have a complex etiology and adolescents’ personal characteristics and psychological functioning should be taken into consideration [11]. First of all, adolescence is a time when the primary causes of fatality are closely associated with personal behavioral choices [10]. For example, impulsivity has been associated with involvement in collisions, particularly in middle adolescence [8] and some authors have highlighted that these individuals operate non-cognitive (for example, affective) cues in their decision-making process while driving [11]. Therefore, in the developmental psychopathology perspective [12], it is of cardinal importance to consider this phenomenon bearing in mind neurobiological, cognitive, and individual psychological factors. Externalizing symptoms have been found to be associated with anxiety [13], which is a factor that increases the possibility of traffic crashes [14], but other evidence supports the fact that internalized symptoms are related to dissociative driving style and fallacies that lead to collisions [15]. There is a vast literature that addresses emotional behavioral functioning of adolescents after the MVC, in terms of psychological repercussions such as post-collision stress symptoms [16], depression [17], anxiety [18], and the importance of the role of family’s response [19]. However, the emotional functioning of adolescents before the collision is far less explored [20] because a certain psychological functioning in adolescents could increase the possibility of being involved in an MVC. Moreover, the majority of the literature tends to consider these variables as outcomes of the MVC [21,22], while, based on other studies [23,24,25], they could also be studied and investigated as predictors of MVCs. For instance, alexithymic traits are known to be related to risk-taking in adolescents [26]. Carbone [27,28] proposes to consider adolescents’ collisions as “acting-outs” determined by a specific psychological maladaptive functioning, and by adolescents’ struggles in expressing and/or recognizing their emotions. Emotional–behavioral functioning is known to be associated with risk-taking behaviors in adolescence. Researchers have hypothesized that adolescence constitutes a particularly relevant moment in the development of psychopathology [23]. Considering this theoretical framework, alexithymia and the use of non-adaptive defense strategies play a major role in risk-taking during adolescence [24]. Especially when adolescents visit the emergency room several times, we could consider this behavior as an unconscious attempt to obtain psychological help [29]. Coherently with recent literature aimed at investigating variables related to the high rates of traffic collisions observed in adolescents [11,23,25], we emphasize the importance of considering the dynamic interplay between factors of different natures, environmental, individual, and relational, placing particular emphasis on the role played by the family and the adolescent’s psychological functioning. Other studies have underlined the importance of family when studying risky behaviors in general and related to driving in adolescence [23,25]. Not only does adequate family communication seem to be of cardinal importance in shaping adolescents’ perception of safety practices in driving (e.g., wearing seatbelts when driving) [30], but it is also important in promoting self-regulation in emotions, protecting adolescents from engaging in risky behaviors [31,32]. A positive family environment could be important in shaping less difficulty in identifying and expressing emotions, which are—in turn—associated with general risky behaviors [33]. In terms of developmental psychopathology, adolescents indulge in risky behaviors because of a developmental drive to perceive themselves as independent and gain authority when compared with their peers [34]. However, studies suggest that when there is a positive family environment, adolescents’ risk-taking behaviors usually decrease [35,36]. Taking these factors into consideration and following recommendations from previous studies [23,24,25], we decided to investigate a group of adolescents that sought help more than three times in a year in different emergency rooms in Italy after being involved in an MVC. We aimed at investigating adolescents’ general emotional–behavioral functioning, the use of defense strategies, alexithymic traits, and the possible contribution of gender and perceived family collaboration. In line with results proposed in other studies [25,28,33,37], we hypothesized that adolescents who repeatedly visited emergency departments due to MVCs over the course of a year would show:

1. A maladaptive psychological functioning, with male adolescents showing higher scores [7];
2. difficulties in identifying and describing their own emotions;
3. a massive presence of immature strategies.

We also hypothesized that perceived family collaboration would have an impact on adolescents’ psychological functioning, on the difficulty in identifying and expressing their own emotions, and on the use of maladaptive defense strategies.

## 2. Materials and Methods

We used a consecutive sampling method to create a convenience sample. We used the following inclusion criteria: (1) no referred psychiatric diagnosis in the subjects; (2) no presence of PTSD symptoms or acute stress at the moment of the study. These symptoms were assessed with the SCID I (Non-Patient Edition [38]); (3) no undergoing of any other medical and/or psychological treatment. We recruited 150 adolescents (N = 150; age range: 14–17 years; male N = 75; female N = 75) who visited an Italian emergency department consequent to an MVC more than 3 times over the course of one year. We excluded from the sample: adolescents who were passengers at the moment of the collision; adolescents who suffered serious injuries; adolescents who did not give consent to participate in the study; adolescents whose parents denied consent for their offspring to participate; adolescents who had a psychiatric diagnosis. Most of the adolescents were Caucasian (93.9%) and 66% of their families had a houshold income between EUR 28.00 and 55.000 per year. Of the adolescents, 78.1% were in intact family groups and 63% of them were primogeniture. None of the anamnestic parts of the questionnaire were mandatory apart from age and gender.

### 2.1. Data Procedure

In line with the Declaration of Helsinki, the Ethical Committee of La Sapienza, University of Rome accepted the present research plan before the start of the present study. All the participants were asked to complete an informed consent document. The anonymity and privacy of any personal information were guaranteed. Participants in this study were given the following self-report tools.

### 2.2. Questionnaires

The Youth Self-report/11-18 (YSR/11-18) [39] is a self-report questionnaire that investigates behavioral and emotional difficulties experienced in the previous 6 months. It is composed of 112 problem items, scored on a 3-point Likert scale (0 = not true; 1 = somewhat or sometimes true; 2 = very or often true). The tool incorporates the following subscales: Withdrawn, Somatic complaints, Anxious/Depressed, Social problems, Thought problems, Attention problems, Delinquent behavior, Aggressive behavior, and Self-destruction identity. The tool has a good internal consistency with Cronbach’s alphas ranging from 0.71 to 0.95.

The Toronto Alexithymia Scale (TAS-20) is a self-report scale composed of 20 items [40,41]. It has a 5-point Likert scale system (1 = not at all true; 5 = totally true). The alexithymia construct is theoretically coherent with a 3-factor structure. Factor 1 is difficulties identifying feelings; Factor 2 is difficulties describing feelings; Factor 3 is external oriented thinking. The higher the score on the scales, the more maladaptive is the psychological functioning. The scale demonstrates good internal consistency and test–retest reliability (the total score’s internal reliability coefficient is 0.86).

The Response Evaluation Measure for Youth (REM-71) [42,43] is a self-report questionnaire composed of 71 items that allows the evaluation of 21 defense mechanisms (Acting out, Splitting, Displacement, Dissociation, Fantasy, Passive aggression, Projection, Repression, Omnipotence, Undoing, Conversion, Somatization, Withdrawal, Suppression, Denial, Humor, Intellectualization, Reaction Formation, Idealization, Altruism, Sublimation). Every defense strategy is individually assessed by 3 or 4 items each and rated on a 9-point scale from “strongly disagree” (1) to “strongly agree” (9). Higher scores on this questionnaire stand for a more massive use of defense strategies. This tool has a 2-factor structure. Factor 1 includes maladaptive defenses with a greater rate of reality distortion leading to less flexible functioning (defenses 16 to 21). The current Italian version showed a good internal consistency and test–retest reliability (reliability coefficient 0.84).

The Perceived Collective Family Scale [44] is a 20-item self-report tool on a 7-point Likert scale (1 = not well at all; 7 = very well). The aim of the tool is to investigate the perceived operative capabilities of the family as a whole, such as managing daily tasks, achieving consensus coping together with unplanned stressors, providing affection, finding pleasure in time spent together. “How well can the family work together to share household responsibilities?” explains assessment of the family operating as a system. We used the global efficacy total score [45].

## 3. Results

### 3.1. Data Analysis

We carried out a series of multivariate analyses of variance (MANOVAs) in Group 1 (females) and Group 2 (males) to assess the adolescents’ psychological functioning. We used MANOVA to assess whether there were significant differences in perceived family collaboration in female and male adolescents. Subsequently, we conducted linear regressions to examine the contribution of perceived family collaboration and group on all of the YSR and TAS-20 subscales, and on Factor 1 and 2 of the REM-71. All analyses were performed with SPSS software (Version 21.0, IBM, Armonk, NY, USA).

#### 3.1.1. Emotional–Behavioral Profiles

Results of the MANOVA yielded that there was not a statistically significant difference between the two groups, female and males, in the combined dependent variables. Based on these results, female and male adolescents did not differ in their emotional–behavioral functioning significantly. However, 25% of our sample (N = 50; m = 25; f = 25) surpassed the clinical cut-off score for the YSR total. These results are shown in Table 1.

#### 3.1.2. Adolescents’ Use of Defense Strategies

Results of the MANOVA yielded that there was not a statistically significant difference between female and male adolescents in the use of maladaptive (Factor 1) and adaptive (Factor 2) defense strategies. Results showed a non-significant interaction effect between gender and defense use, but there were two significant main effects regarding the use of particular defense strategies (Wilk’s lambda = 0.006; F = 846.64; *p* < 0.000). Specifically, the Bonferroni post hoc test confirmed that female adolescents showed significantly higher scores on Withdrawn (F = 4.621; *p* < 0.03) while male adolescents reported significantly higher scores on Suppression (F = 4.191; *p* = 0.04). Of our sample, 83.3% exceeded the clinical cut-off for Factor 1 in the REM-71 [46]. These results are shown in Table 2.

#### 3.1.3. Female and Male Adolescents’ Alexithymic Traits

Results of the MANOVA yielded that there was not a statistically significant difference between the two groups, female and males, in the combined dependent variables. Based on these results, female and male adolescents do not differ in their alexithymic traits significantly. However, 31.3% of our sample (N = 47; m = 23; f = 24) exceeded the clinical cut-off for the TAS-20 total score (>61). These results are shown in Table 3.

#### 3.1.4. Female and Male Adolescents’ Perceived Family Collaboration

Results of the ANOVA yielded that there was not a statistically significant difference between the two groups, females and males, in our dependent variable. Based on these results, female and male adolescents did not differ in their perceived family support significantly. These results are shown in Table 4

#### 3.1.5. Contribution of Perceived Family Collaboration to Adolescents’ Emotional–Behavioral Profiles

We then conducted linear regressions to verify the possible contribution of perceived family collaboration to adolescents’ emotional–behavioral functioning, their difficulty in identifying and describing their emotions, and their use of more maladaptive defense strategies. Group belonging (gender) and perceived family collaboration were used as predictors, while self-report scores on YSR, TAS-20, and REM-71 were computed as regressors. Gender showed no significant effect on any of our tools’ scores. The higher the perceived family collaboration was, the lower the scores on all the subscales of the YSR were (except for the subscale of Attention problems). In other words, a good perceived family collaboration predicted a more adaptive emotional–behavioral functioning both for female and male adolescents. These results are shown in Table 5.

Moreover, higher scores in perceived family collaboration predicted lower scores on the three TAS-20 subscales. In other words, for both female and male adolescents, a good perceived family collaboration predicts a greater capacity in identifying and expressing their own emotion and less external oriented thinking. These results are shown in Table 6.

Lastly, higher scores in perceived family collaboration predicted lower scores on Factor 1 of the REM-71, meaning that a good family collaboration predicts a less frequent use of maladaptive defense strategies. These results are shown in Table 7.

## 4. Discussion

The purpose of this study was to investigate the general emotional–behavioral functioning, the use of defense strategies, alexithymic traits, and the possible role of gender in female and male adolescents without a psychiatric diagnosis who experienced more than three motor vehicle collisions (MVCs) in one year. Further, we intended to verify whether a low family collaboration (as perceived by adolescents) could contribute to their own maladaptive psychological functioning. For what concerns our first aim, in line with other studies [10,11], we expected male adolescents to show more maladaptive emotional–behavioral profiles. However, our sample did not show a significant difference between male and female adolescents. We also have to notice that—in our sample—only 25% of the subjects exceeded the cut-off and could be considered clinically relevant for a psychopathological risk. However, in line with our second aim, 83.3% of our adolescents surpassed the clinical cut-off for Factor 1 of the REM-71, meaning that these adolescents are making greater use of primitive and more maladaptive defense strategies. We speculate that adolescents who experience multiple MVCs and who routinely use emergency departments do not necessarily show clinically relevant psychopathological profiles. Therefore, we may speculate that these incidents are not the direct outcome of severe psychopathological profiles; rather, they could be considered unconscious acting-outs aimed at seeking help for a psychological sufferance which takes the form of a collision. Male and female adolescents seem to return to the same defense strategies, except for Withdrawal, which seems to be used by male adolescents more. Both these defense strategies are considered maladaptive strategies and this gender difference is coherent with other literature [42]. As Cramer [47] points out, female and male adolescents are more likely to use—respectively— feminine and masculine defense strategies. Suppression can be considered a “masculine” defense strategy, while withdrawal can be, in turn, considered “feminine”. However, we should be cautious when interpreting this fact. The relative paucity of our sample does not allow us to derive more robust explanations and this difference in the use of these two defense strategies could be caused by a number of factors, first among them the unconscious internalization of gender stereotypes [45,48]. Our main point, for what concerns the importance of defense strategies, is well summarized in the recent work by Carbone et al. [27,28]. Adolescents’ psychological functioning and use of maladaptive defense strategies deriving from difficulties in emotional regulation preceding an MVC could increase the likelihood of a collision. Therefore, from our perspective, collisions in adolescence might not always be considered as a result of distraction or ignorance of road safety regulations, but rather as expressions of psychological distress that the adolescent is unaware of [28]. In this framework, resorting more than three times a year to emergency rooms due to minor or major collisions can thus be interpreted as a cry for psychological help. For what concerned our last aim, perceived family collaboration seems to have an impact on adolescents’ psychological functioning, on the difficulty of identifying and expressing their own emotions, and on the use of maladaptive defense strategies both in female and male adolescents. It is well known in the literature that family functioning is of cardinal importance in shaping adolescents’ mental health. Adolescence is a phase of cognitive and neurobiological development, psychosocial challenges, and continuous changes in emotional regulation mechanisms to manage stressors caused by new situations [49]. Difficulties in family functioning starting in childhood are often related to a more maladaptive behavioral functioning in adolescence and in adulthood [50]. Emotional–behavioral functioning is also associated with risk-taking behaviors and could explain the greater exposure of these adolescents to MVCs. On the contrary, a well-functioning family could constitute a protector factor against adolescent involvement in risky situations [31]. The perceived collaboration and collaborations of the parents themselves reduce or increase the adolescents’ tendency to indulge in perilous situations and their maladaptive psychological functioning in general. The majority of the literature has focused on the importance of family functioning in terms of an aspect which is mostly related to treatment outcomes after the collision, in particular as a mediator or a risk or protective factor for the forming of post-traumatic and stress-related symptoms [51,52]. The strength of this study is considering family functioning as an important before factor that can play a cardinal role in predicting adolescents’ risk behaviors related to MVCs. However, our study has some limitations. First, the race and socioeconomic statuses’ homogeneity as well as the relatively small sample size do not enable wide generalizations of our results. Second, we did not assess the quality of peers’ relationships that can be an important risk or protective factor associated with risky behaviors in adolescence as much as family functioning can be [53]. Third, we only used a self-report questionnaire to investigate adolescents’ psychological profiles and perceived family collaboration. Moreover, our study is descriptive. We cannot statistically demonstrate which of these factors contribute to the phenomenon. Future research should focus on the understanding of the psychological factors that affect risky behavior when driving that potentially lead to MVCs in adolescence, particularly taking into consideration the role of personality traits, alexithymia, and the role of the family which have not been sufficiently explored. Notwithstanding the above limitations, these findings may be useful for mental health professionals when planning prevention programs for adolescents focusing on their ability of identifying and expressing their emotions and on the use of more adaptive defense strategies while dealing with new and risky situations that could potentially cause stress. Emergency rooms could integrate screening processes when these adolescents seek help after an MVC, in order to redirect them to local and public services that offer the psychological help they are seeking. Moreover, as an interesting strand of literature points out, these intervention programs should be integrated with the empowerment of perceptual and motor skills starting from childhood, which have been related to safer driving practices during adolescence and adulthood [54].

## 5. Conclusions

Recent contributions in the field of adolescents’ risk-taking have underlined that emotional–behavioral functioning, alexithymic traits, the massive use of maladaptive defense strategies, and a low perceived family collaboration represent crucial risk factors for adolescents’ MVCs. However, the majority of studies focus on the psychological outcomes after the collisions. To date, few studies [23,24,25] have yet explored the complexity of the psychological functioning of adolescents who repeatedly access an emergency department due to MVCs, suggesting the importance of implementing a more vast knowledge of this phenomenon in order to make the planning of preventive programs more effective. Overall, our findings further supported the emerging importance of understanding the psychological functioning of these adolescents before MVCs in order to implement prevention programs aimed at supporting their ability to identify and express their emotions, to use more adaptive defense strategies, and to work with the families in order to create a more open communication that can significantly reduce adolescents’ risky behaviors related to driving [55].

## Figures and Tables

**Table 1:** Mean scores, standard deviations, and *p* values for adolescent scores on the YSR scales.

	M Mean (SD)	F Mean (SD)	*p*
Withdrawn	6.46 (2.9)	5.89 (3.2)	0.25
Somatic complaints	7.32 (2.8)	7.18 (2.5)	0.75
Anxious–depressed	11.28 (8.0)	10.45 (7.2)	0.51
Social problems	5.86 (2.3)	5.62 (2.4)	0.53
Thought problems	4.56 (1.6)	4.68 (1.4)	0.62
Attention problems	3.61 (1.26)	3.41 (1.1)	0.32
Delinquent behavior	3.22 (1.1)	3.55 (1.1)	0.14
Aggressive behavior	15.05 (7.5)	14.74 (7.6)	0.81
Self-destructive behavior	12.57 (4.0)	12.17 (4.4)	0.56
Internalizing symptoms	19.85 (11.0)	20.24 (11.2)	0.46
Externalizing symptoms	18.03 (12.2)	18.66 (11.8)	0.83
Total	41.05 (30.5)	42.17 (30.5)	0.75

T-score cut-offs: 65 to 69 = borderline, 70+ = clinical; no T-score > 100 or <50 generated for narrow band scales.

**Table 2:** Mean scores, standard deviations, and *p* values for adolescent scores on the REM-71 scales.

	M Mean (SD)	F Mean (SD)	*p*
Acting out	4.88 (1.3)	5.01 (1.21)	0.53
Splitting	7.15 (8.6)	7.16 (9.1)	0.98
Displacement	4.77 (1.2)	4.68 (0.9)	0.59
Dissociation	5.30 (1.3)	5.42 (1.4)	0.61
Fantasy	6.11 (6.3)	5.58 (5.2)	0.57
Omnipotence	5.11 (0.7)	5.28 (0.7)	0.18
Passive aggression	5.66 (1.1)	5.59 (1.1)	0.67
Projection	4.42 (1.4)	4.54 (1.4)	0.62
Repression	4.99 (1.5)	5.11 (1.4)	0.68
Undoing	5.95 (1.3)	6.05 (1.2)	0.65
Sublimation	5.24 (1.3)	5.31 (1.3)	0.86
Conversion	2.20 (1.1)	2.23 (1.4)	0.41
Somatization	4.26 (1.5)	4.06 (1.4)	0.11
Withdrawal	5.78 (1.2)	15.71 (7.2)	0.03
Altruism	6.71 (1.1)	7.12 (1.2)	0.95
Denial	4.61 (1.1)	4.61 (0.8)	0.81
Humor	5.18 (1.2)	5.44 (1.1)	0.12
Idealization	6.04 (1.1)	6.12 (1.1)	0.75
Intellectual	5.63 (0.9)	5.62 (1.1)	0.67
Reaction formation	4.43 (0.8)	4.39 (0.9)	0.08
Suppression	4.52 (1.1)	4.71 (0.8)	0.04
Factor 1	5.56 (5.2)	5.48 (3.3)	0.67
Factor 2	5.58 (1.2)	5.61 (1.2)	0.91

Cut-off: mean score of 4.40.

**Table 3:** Mean scores, standard deviations, and *p* values for adolescent scores on the TAS-20 scales.

	M Mean (SD)	F Mean (SD)	*p*
Difficulty identifying feelings	17.55 (7.6)	18.05 (8.1)	0.65
Difficulty describing feelings	9.9 (2.7)	10.1 (2.6)	0.87
External oriented thinking	22.23 (8.1)	22.37 (7.8)	0.91
Total	49.25 (14.5)	51.08 (13.7)	0.34

Cut-off: <51 = no alexithymia; 52 to 60 = possible alexithymia; >61 = clinical alexithymia.

**Table 4:** Mean scores and standard deviations for female and male adolescent perceived family collaboration.

	M Mean (SD)	F Mean (SD)
Perceived Family Collaboration	47.93 (17.9)	46.74 (18.3)

**Table 5:** Contribution of perceived family collaboration to YSR subscales: R^2^, beta, t and *p* values.

YSR Subscales	Perceived Family Collaboration			
	R^2^	Beta	t	*p*
Withdrawn	0.514	−0.717	−12.500	<0.001
Somatic complaints	0.506	−0.711	−12.300	<0.001
Anxious–depressed	0.664	−0.815	−17.093	<0.001
Social problems	0.372	−0.610	−9.35	<0.001
Thought problems	0.105	−0.325	−4.175	<0.001
Attention problems	0.026	0.162	1.995	0.32
Delinquent behavior	0.198	−0.445	−6.048	<0.001
Aggressive behavior	0.657	−0.811	−16.841	<0.001
Self-destructive behavior	0.463	−0.680	−11.296	<0.001
Internalizing symptoms	0.772	−0.879	−22.386	<0.001
Externalizing symptoms	0.774	−0.880	−22.532	<0.001
Total	0.814	−0.902	−25.416	<0.001

**Table 6:** Contribution of perceived family collaboration to REM-71 subscales: R^2^, beta, t and *p* values.

TAS-20	Perceived Family Collaboration			
	R^2^	Beta	t	*p*
Difficulty identifying feelings	0.724	−0.851	−19.695	<0.001
Difficulty describing feelings	0.085	−0.292	−3.712	<0.001
External oriented thinking	0.612	−0.782	−15.285	<0.001
Total	0.139	−0.373	−4.885	<0.001

**Table 7:** Contribution of perceived family collaboration to REM-71 factors: R^2^, beta, t and *p* values.

REM-71	Perceived Family Collaboration			
	R^2^	Beta	t	*p*
Factor 1	0.000	0.017	0.210	<0.001
Factor 2	0.270	−0.519	−7.395	<0.001

## Data Availability

Data are available on request to the corresponding author.
